# Banff Human Organ Transplant Consensus Gene Panel for the Detection of Antibody Mediated Rejection in Heart Allograft Biopsies

**DOI:** 10.3389/ti.2023.11710

**Published:** 2023-09-04

**Authors:** Alessia Giarraputo, Guillaume Coutance, Olivier Aubert, Marny Fedrigo, Fariza Mezine, Dina Zielinski, Michael Mengel, Patrick Bruneval, Jean-Paul Duong van Huyen, Annalisa Angelini, Alexandre Loupy

**Affiliations:** ^1^ Université Paris Cité, INSERM U970 PARCC, Paris Institute for Transplantation and Organ Regeneration, Paris, France; ^2^ Cardiovascular Pathology, Department of Cardiac, Thoracic, Vascular Sciences and Public Health, University of Padua, Padua, Italy; ^3^ Department of Cardiac and Thoracic Surgery, Cardiology Institute, Pitie Salpetriere Hospital, Assistance Publique-Hopitaux de Paris (AP-HP), Sorbonne University Medical School, Paris, France; ^4^ Department of Kidney Transplantation, Necker Hospital, Assistance Publique—Hôpitaux de Paris, Paris, France; ^5^ Department of Laboratory Medicine and Pathology, Faculty of Medicine and Dentistry, University of Alberta, Edmonton, AB, Canada; ^6^ Pathology Department, Hôpital Necker, AP-HP and Université de Paris, Paris, France

**Keywords:** antibody-mediated rejection, heart transplantation, gene expression, transcriptome, heart rejection, molecular profiling

## Abstract

The molecular refinement of the diagnosis of heart allograft rejection based on whole-transcriptome analyses faces several hurdles that greatly limit its widespread clinical application. The targeted Banff Human Organ Transplant gene panel (B-HOT, including 770 genes of interest) has been developed to facilitate reproducible and cost-effective gene expression analysis of solid organ allografts. We aimed to determine *in silico* the ability of this targeted panel to capture the antibody-mediated rejection (AMR) molecular profile using whole-transcriptome data from 137 heart allograft biopsies (71 biopsies reflecting the entire landscape of histologic AMR, 66 non-AMR control biopsies including cellular rejection and non-rejection cases). Differential gene expression, pathway and network analyses demonstrated that the B-HOT panel captured biologically and clinically relevant genes (IFNG-inducible, NK-cells, injury, monocytes-macrophage, B-cell-related genes), pathways (interleukin and interferon signaling, neutrophil degranulation, immunoregulatory interactions, endothelial activation) and networks reflecting the pathophysiological mechanisms underlying the AMR process previously identified in whole-transcriptome analysis. Our findings support the potential clinical use of the B-HOT-gene panel as a reliable proxy to whole-transcriptome analysis for the gene expression profiling of cardiac allograft rejection.

## Graphical Abstract

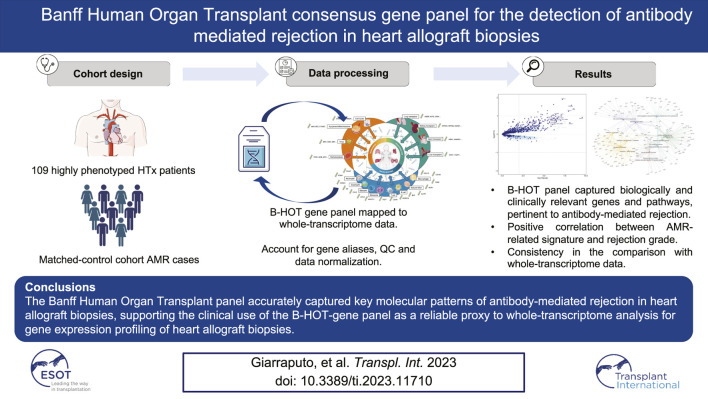

## Introduction

Allograft rejection remains an important complication after heart transplantation associated with poor outcomes. While the incidence and clinical importance of acute cellular rejection has declined over time, antibody-mediated rejection (AMR) is now recognized as a major risk factor for patient death, graft loss and various allograft injuries [1]. Even if important advances have been made in the standardization of its pathology diagnosis, disease severity, degree of myocardial injury and progression stage are crucial pieces of information which are poorly captured by the current working formulation [1]. Whole-transcriptome (WT) gene expression analysis of myocardial tissue has been shown to be a relevant companion tool to refine the pathology diagnosis of AMR after heart transplantation [2, 3]. However, important drawbacks have limited its widespread clinical application (extra-core sampling and inherent procedural risks, low reproducibility, technical and analytical burden) [4]. Targeted molecular profiling applicable to formalin-fixed paraffin-embedded (FFPE) endomyocardial biopsies (EMB) may allow the implementation of molecular diagnosis into the clinical routine [5]. Recently, the Banff Human Organ Transplant Panel (B-HOT), a consensual targeted panel comprising 770 genes, has been designed to capture molecular expression related to tissue injury, innate and adaptive immunity and rejection in solid organ transplants in order to facilitate cost-effective and reproducible expression analysis of solid organ allografts [5]. The combination of a FFPE-based tissue assessment together with pathological phenotyping of heart allograft biopsies had the potential of enlightening novel pathological mechanisms involved in antibody-mediated rejection correlating with pathological assessment. Whether this targeted panel provides enough granularity to capture the complexity and heterogeneity of AMR compared to whole-transcriptome analysis still remains unknown. We aimed to analyze *in silico* the ability of the B-HOT panel to capture relevant genes, pathways and networks associated with AMR compared to the whole-transcriptome analysis.

## Methods

### Study Design and Participants

The study cohort consisted of 137 heart transplant biopsies from 109 patients performed between 2006 and 2011 at four French referral institutions (Hôpital Georges Pompidou and Pitié Salpétrière in Paris, Hôpital Laennec in Nantes, and Hôpital Charles Nicolle in Rouen), that have been previously studied and published [6]. This study is a non pre-specified ancillary analysis of a prospective study. This cohort comprised patients with AMR, ACR and non-rejection related cases. This study was conducted in compliance with the Declaration of Helsinki and approved by the institutional review board (CPP Île de France II - protocol 2014-12-26, registration number: 00001072).

### Definition of Antibody-Mediated Rejection of Heart Allografts

Histology of EMBs was assessed by 2 expert pathologists (PB and JPVDH). Biopsies were graded according to the most recent international working formulations of Society for Heart and Lung Transplantation [1, 7]. As recommended, immunohistochemistry based on C4d capillary deposition (positive if >50% of the capillaries were labeled) and/or CD68-positive staining (positive if intravascular CD68^+^ macrophages were present in >10% of the capillaries) were evaluated.

### Histological, Immunohistochemical and Transcriptomic Phenotyping of Biopsies

C4d staining was performed by immunohistochemistry on paraffin sections using an immunoperoxidase method and an anti-C4d antibody, additional staining were performed for characterization of macrophages capillary infiltration (anti-CD68) [8, 9]. All biopsies were processed for whole-transcriptome analysis. RNA extraction, labeling, and hybridization were performed to the HG-U219 GeneChip arrays (Affymetrix, CA, USA) following manufacturer’s protocols (www.affymetrix.com). Microarrays were scanned using the Affymetrix Gene Array Scanner, generating.cel files with the GeneChip Operating Software Version 1.4.0 (Affymetrix) as previously described [10].

### Mapping Banff Human Organ Transplant (B-HOT) Genes to Array Probesets

B-HOT gene annotations were defined according to the Banff 2019 meeting report [5]. The B-HOT panel consists of 770 genes, including 12 housekeeping genes only used for quality control and data normalization, and 758 endogenous genes to which microarray gene symbols were assigned. We excluded 4 viral-related genes (BK VP1, BK large T Ag, CMV UL83, EBV LMP2) due to lack of microarray correspondence. We corrected and accounted for gene alias discrepancies, noticing 4 endogenous genes that could not be mapped to the array: IGHG4, MIR155HG, OR2I1P, TRDC. Microarray gene annotations were retrieved from Bioconductor (hgu219.db) and mapped to probeset IDs, with multiple gene annotations being mapped to the same probeset ID. We finally excluded control array probeset AFFX and ERCC, resulting in unique annotated probesets mapping to relative genes.

### Differential Expression Analysis

Raw gene expression data were normalized using the Robust Multichip Average (RMA) expression measure algorithm. Low variance probesets were excluded using IQR<0.5 filtering, with a total of 24697 probesets left. Differential expression analysis was conducted by fitting a linear model to the normalized expression values for each probeset. Fold changes and t-statistics were computed for the contrast of interest AMR versus non-AMR biopsies. Standard errors were moderated using an empirical Bayes model to compute a moderated t-statistic and a log-odds of differential expression for each contrast and each probeset [11]. Probesets were collapsed by gene identifiers for a total of 12170 unique genes, 662 of which were in the B-HOT panel, then by lowest *p*-value and highest fold changes (in the event of a *p*-value tie), adjusting nominal *p*-values for multiple comparisons using the Benjamini-Hochberg method.

We then compared differential expression analysis results derived from the whole-transcriptome or the targeted genes panel. Significant genes associated with AMR were filtered according to a false discovery rate *p*-value lower than 0.05 and annotated according to Uniprot as well as GeneCards databases [12, 13].

### Analysis of AMR-Associated Pathways and Networks Based on B-HOT Genes

The enrichment pathways were generated based on the differentially expressed genes (FDR <0.05) for the contrast of interest derived from whole-transcriptome genes or restricted to B-HOT genes using ReactomePA [14]. Pathophysiological categories were then combined to investigate gene-to-gene interconnection by cnet plots (*enrichplot package*). Hierarchical clustering of enriched terms was implemented to account for pairwise similarities using Jaccard similarity index [15]. Statistical analyses were performed using R software (version 4.0.5).

## Results

### Characteristics of Patients and Biopsies

Patients’ characteristics (137 biopsies included from 109 patients from 4 French referral centers) are shown in Supplementary Table S1. The patients were mostly man (68.8%), their mean age at transplant was 43.2 years. A vast majority of biopsies were protocol biopsies (85%). The median biopsy time relative from transplant time is 10.67 months (IQR = 34.7). Among 137 heart allograft biopsies included, histology-based diagnosis identified 71 biopsies reflecting the entire spectrum of AMR as defined by international working formulations (pAMR1(I+): *n* = 20, pAMR1(H+): *n* = 24; pAMR2/3: *n* = 27) and 66 biopsies without AMR (comprising 24 with acute cellular rejection (ACR) and 42 with non-rejection diagnoses).

### B-HOT Panel Gene Expression Appraisal to Detect AMR

**FIGURE 1:**
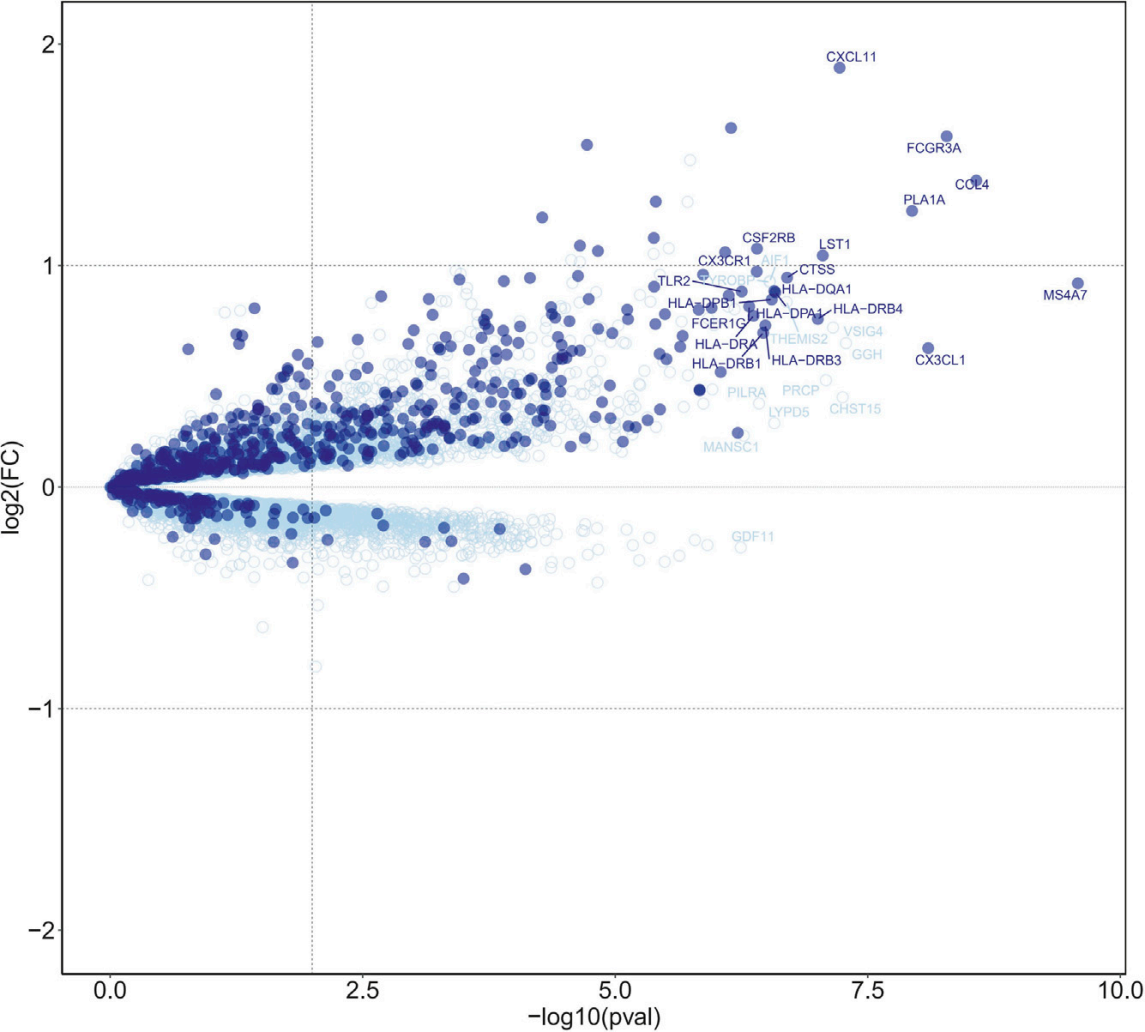
Differential expression analysis of antibody-mediated rejection, highlighting B-HOT panel genes. Volcano plot of differentially expressed genes associated with AMR in heart allografts. Each dot represents an individual transcript. Dark blue points indicate genes targeted in the B-HOT panel and light blue points represent whole-transcriptome genes included on the microarray. The top 30 ranked differentially expressed gene symbols are shown according to 0.05 threshold (false discovery rate adjusted *p*-values). Differentially expressed B-HOT-related genes are associated to: IFNG-inducible genes (CX3CL1, CXCL11, HLA-DRB4, HLA-DRB1, HLA-DRB3, HLA-DPA1, HLA-DRA, HLA-DQA1, HLA-DPB1); NK-cell (CCL4, FCGR3A, CX3CR1); Injury (PLA1A, CTSS); Monocytes-macrophage (MS4A7, LST1, CSF2RB, TLR2); B-cell associated (FCER1G).

B-HOT panel reliability in detecting gene expression pattern associated with AMR was evaluated through the comparison of the global gene expression changes in biopsies diagnosed with antibody-mediated rejection (*n* = 71) compared to all biopsies without AMR (n = 66), considering whole transcriptome genes or only those included in the targeted panel. The differential expression analysis showed a high enrichment of B-HOT related genes (Figure 1). Of the top 30 genes identified in the whole transcriptome analysis, 19 were included in B-HOT panel and covered major immune response functions and cell specific types related to AMR: IFNG-inducible genes and adaptive immune response (CX3CL1, CXCL11, HLA-DRB4, HLA-DRB1, HLA-DRB3, HLA-DPA1, HLA-DRA, HLA-DQA1, HLA-DPB1); NK-cell related (CCL4, FCGR3A, CX3CR1); Injury related genes (PLA1A, CTSS); Monocytes-macrophage genes (MS4A7, LST1, CSF2RB, TLR2); B-cell associated gene (FCER1G) [12, 13]. We found a strong positive correlation between AMR-related gene expression and increasing AMR rejection grade (Supplementary Figure S1). The remaining 11 genes were related to functions and cell components associated with an unspecific immune response (Supplementary Table S2),: GGH (metabolism, hydrolysis); CHST12 (protein transport); VSIG4 (phagocytic receptor, negative regulator for T-cell receptor signaling and IL-2); PRCP (lysosomal peptidases); THEMIS2 (T-cell receptor signaling); LYPD5 (extracellular region protein); AIF1 (actin-binding protein, induced by cytokine and interferon); TYROBP (adapter protein); PILRA (cellular inhibitory receptor); MANSC1 (membrane protein); GDF11 (mediate cell differentiation, secrete ligand of TGF-beta).

### Pathway and Gene-Concept Network Analysis of B-HOT AMR-Associated Genes

**FIGURE 2:**
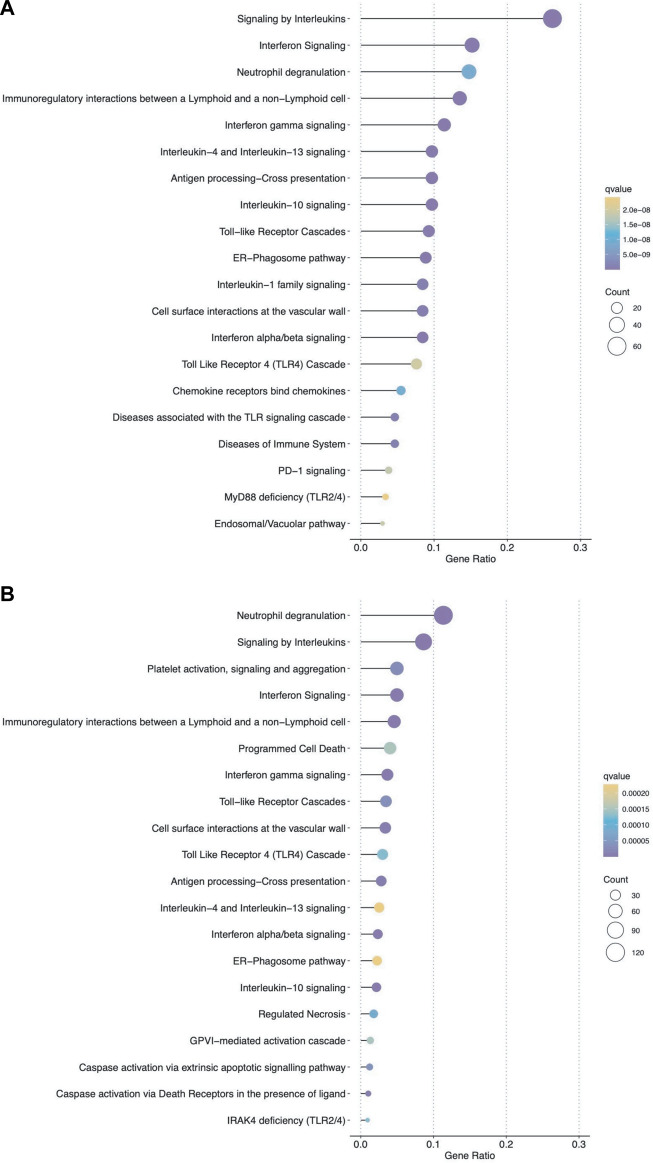
Top ranked pathways associated with significant genes for antibody-mediated rejection using either the targeted panel only or all microarray genes. Dot plots show the top 20 enriched pathways based on significant differentially expressed genes (false discovery rate <0.05) associated with AMR in heart allografts (Panel **(A)**: B-HOT genes; Panel **(B)**: WT genes). The *x*-axis represents different gene categories, each enrichment result is plotted in accordance with the gene ratio (number of genes associated with the given pathway divided by the total number of genes analyzed). The size of the dots represents the number of genes in the significant differentially expressed gene list associated with the pathway and the color intensity of the dots represents the false discovery rate adjusted *p*-value.

**FIGURE 3:**
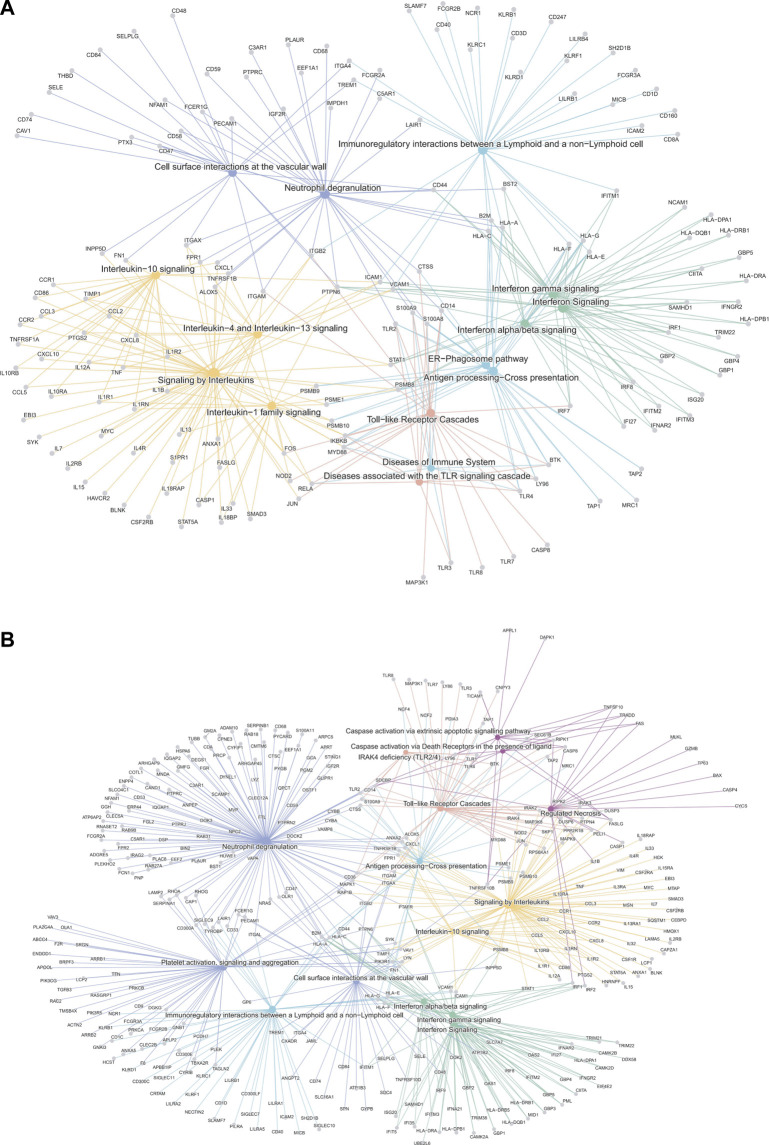
Gene-Concept network analysis of significant differentially expressed genes associated with antibody-mediated rejection using the targeted panel or whole-transcriptome genes. The interaction networks depict the gene-to-gene interconnection in the enriched terms of biological categories from the Reactome repository. The top-ranked 15 signaling pathways are shown according to the general class of associated pathophysiological events involved in AMR (derived from B-HOT only and WT genes). Node size refers to the number of differentially-expressed genes in the enriched pathway. Genes shared between edges refer to terms belonging to multiple pathophysiological categories. Network plots of AMR associated genes (Panel **(A)**: B-HOT genes; Panel **(B)**: WT genes). Several pathways were shared between the two gene sets, especially related to: Toll-like receptor cascade, Interleukin and Interferon signaling. The network derived from B-HOT genes included pathways more specific to AMR pathophysiology associated with activation of specific Interleukin (IL-1, IL-4 and IL-13) and antigen processing cross-presentation mechanisms (ER-mediated). Networks based on WT genes showed categories with less specificity for antibody-mediated response, including Caspase activation and Apoptotic signals.

We then analyzed the ability of the B-HOT panel to capture clinically relevant biological pathways involved in AMR using functional enrichment analysis of all significant differentially expressed genes (FDR<0.05). Major pathophysiological mechanisms related to antibody-mediated response and injury (Figures 2A, B) identified in the whole-transcriptome analysis were also identified in the B-HOT derived analysis: interleukin (q = 1.00E-29) and interferon-gamma (INFG, q = 1.18E-21) signaling, antigen processing cross-presentation (q = 2.86E-15) and neutrophil degranulation (q = 9.03E-09). The whole-transcriptome analysis identified additional categories related to non-specific immune responses including caspase activation and regulated necrosis (Supplementary Tables S3, S4). Finally, we elucidated gene-to-gene interconnections by building functional networks (Figures 3A, B). Targeted- and whole-transcriptome-derived networks showed high interconnections with a large overlap of pathophysiological categories: interleukin signaling, interferon signaling, adaptive immune system, Toll-like receptor cascade and cell surface interactions. Whole-transcriptome-based networks identified additional categories related to homeostasis, apoptosis regulation and caspase activation. Hierarchical clustering of enriched terms highlighted the importance of major immune-related classes in both approaches, displaying organization and relationships between the enriched pathways terms, ranking biological processes and pathways that are relevant to AMR condition (Supplementary Figures S2A, B). Overall, the analysis of pathophysiological mechanisms, gene-to-gene interactions within and between pathophysiological categories, as well as hierarchical clustering demonstrated that the B-HOT panel conserved similar functional information, thus showing less redundancy compared to the whole-transcriptome ones (Figure 3).

## Discussion

In this study, we evaluate the B-HOT panel ability to capture the key features of the molecular signature of AMR through comparative analysis with the whole-transcriptome approach. Using differential expression, pathway, and network analysis, we demonstrate that the B-HOT panel captured clinically-relevant AMR associated genes in heart allografts, and that the derived enriched pathways and functional networks were highly comparable to the whole-transcriptome derived ones. Our results suggest that the targeted panel may be sufficient and sensitive enough to serve as a surrogate to whole-transcriptome analysis.

In the last decade, whole-transcriptome based expression profiling has described the signature of allograft rejection in solid organ transplantation, and allowed the development of predicting models with good performance metrics [6, 16, 17]. However, this approach still relies on extra-biopsy cores, thus limiting the direct correlation of the molecular findings with the histology assessment.

While the molecular refinement of the diagnosis of rejection based on whole-transcriptome approaches faces several hurdles that limit its clinical application (e.g., variation due to cDNA conversion, amplification, labeling, probe redundancy), FFPE-based technology combined with a targeted panel has the potential to reduce experimental complexity, cost and turn-around time, thus refining rejection diagnosis and therapeutic decision-making in the framework of histo-molecular data contextualization. A first attempt to assess the B-HOT panel utility as a proxy for whole-transcriptome profiling on publicly available renal allograft expression data has been reported recently [18]. Our study extends this concept by contextualizing the clinical relevance of the targeted panel in the field of heart transplantation and appraising the gene expression molecular signature with the pathophysiological mechanisms associated with AMR. This advancement offers a more practical and cost-effective method to refine the pathology diagnosis of antibody-mediated rejection, paving the way for its potential implementation into clinical routine and aiding in the understanding of the complex mechanisms underlying heart allograft rejection. Some limitations of the study should be noted. First, additional studies investigating cellular rejection are required to validate the B-HOT panel as a relevant surrogate of whole-transcriptome analysis across the full spectrum of heart transplant pathology. Second, the interest of B-HOT-based molecular diagnostics in clinical practice remains to be evaluated by deriving and validating a specific targeted molecular signature of cardiac allograft rejection in multicenter cohorts. Novel precision diagnostic systems such as the B-HOT panel FFPE-tissue based had the potential to improve diagnostic accuracy, while reducing complexity and turn-around time. Additional studies are needed not only to precise the clinical value of the targeted gene expression analysis but also to combine invasive and non-invasive testing in the clinical field [19]. A synergistic approach that integrates multimodal assessment with multi-disciplinary expertise has never been more important for the global management of heart transplant recipients to optimize therapeutic decision-making and improve patients outcomes [5, 19, 20].

The Banff Human Organ Transplant panel accurately captured key molecular patterns of antibody-mediated rejection in heart allograft biopsies. Our study suggests that this specific targeted panel could be used as a proxy to whole-transcriptome profiling -based analysis after heart transplantation.

## Data Availability

The data that support the findings of this study are available from the corresponding author upon reasonable request. Requests to access these datasets should be directed to AL, alexandre.loupy@inserm.fr.

## Abbreviations

ACR, Acute Cellular Rejection; AMR, Antibody-mediated rejection; B-HOT, Banff Human Organ Transplant Panel; EMB, Endomyocardial Biopsies; FDR, False Discovery Rate; FFPE, Formalin-Fixed and Paraffin-Embedded; HTx, Heart transplantation; INFG, Interferon-gamma; WT, Whole-transcriptome.
