# Supplementary material for: Banff Human Organ Transplant Consensus Gene Panel for the Detection of Antibody Mediated Rejection in Heart Allograft Biopsies
Source: Transpl Int. 2023 Sep 4;36:11710. doi: 10.3389/ti.2023.11710 (PMC10515212; doi:10.3389/ti.2023.11710)
Supplement: Supplementary file 1 [file DataSheet1.docx]

Supplementary material

**Supplementary Table 1.** Characteristics of heart transplant recipients and donors (n=109).

|  | **N*** | **Total** |
| --- | --- | --- |
| **Recipient characteristics** |  |  |
| Recipient age, mean (SD) | 109 | 43.27 (15.38) |
| Recipient gender, n (%) male | 109 | 75 (68.80) |
| **Primary heart disease, n (%)** | 108 |  |
| Valvular cardiomyopathy |  | 6 (5.55) |
| Congenital cardiomyopathy |  | 12 (11.11) |
| Coronary disease |  | 32 (29.63) |
| Myopathy |  | 57 (52.78) |
| Retransplantation |  | 1 (0.93) |
| **Donor characteristics** |  |  |
| Donor age, mean (SD) | 109 | 41.06 (15.00) |
| Donor gender, n (%) male | 109 | 74 (67.89) |

Abbreviations: SD (Standard). *Refers to the number of patients without missing value.

**Supplementary Table 2.** Top 30 significant genes associated with antibody-mediated rejection (AMR) in heart allografts. The B-HOT column indicates inclusion in the panel. EntrezID column refers to unique gene’s identifiers.

**Supplementary Table 3.** Top 30 significant pathways associated with AMR on the B-HOT panel. Enriched pathways are retrieved from Reactome repository, we grouped redundant categories in accordance with physiological function associated. Adjusted -values (referred as q-values) depict significant enrichment from the targeted panel.

| **General Class** | **Top 30 network derived from BHOT genes** | **Reactome identifier** | **q-value** |
| --- | --- | --- | --- |
| Interleukin Signaling | Signaling by Interleukins | R-HSA-449147 | 1.00E-29 |
| Interleukin Signaling | Interleukin-10 signaling | R-HSA-6783783 | 5.57E-24 |
| Adaptive Immune System | Immunoregulatory interactions between a Lymphoid and a non-Lymphoid cell | R-HSA-198933 | 1.00E-22 |
| Interferon Signaling | Interferon gamma signaling | R-HSA-877300 | 1.18E-21 |
| Interferon Signaling | Interferon Signaling | R-HSA-913531 | 2.81E-21 |
| Interferon Signaling | Interferon alpha/beta signaling | R-HSA-909733 | 2.63E-15 |
| Antigen processing-Cross presentation | Antigen processing-Cross presentation | R-HSA-1236975 | 2.86E-15 |
| Interleukin Signaling | Interleukin-4 and Interleukin-13 signaling | R-HSA-6785807 | 4.87E-15 |
| Antigen processing-Cross presentation | ER-Phagosome pathway | R-HSA-1236974 | 1.38E-14 |
| Toll-like Receptor Cascade | Toll-like Receptor Cascades | R-HSA-168898 | 1.59E-10 |
| Endothelial activation | Cell surface interactions at the vascular wall | R-HSA-202733 | 6.80E-10 |
| Toll-like Receptor Cascade | Diseases of Immune System | R-HSA-5260271 | 9.19E-10 |
| Toll-like Receptor Cascade | Diseases associated with the TLR signaling cascade | R-HSA-5602358 | 9.19E-10 |
| Interleukin Signaling | Interleukin-1 family signaling | R-HSA-446652 | 9.19E-10 |
| Innate immune system | Neutrophil degranulation | R-HSA-6798695 | 9.03E-09 |
| Chemokine signaling | Chemokine receptors bind chemokines | R-HSA-380108 | 9.79E-09 |
| Costimulation by the CD28 family | PD-1 signaling | R-HSA-389948 | 1.82E-08 |
| Antigen processing-Cross presentation | Endosomal/Vacuolar pathway | R-HSA-1236977 | 1.88E-08 |
| Toll-like Receptor Cascade | Toll Like Receptor 4 (TLR4) Cascade | R-HSA-166016 | 1.96E-08 |
| Toll-like Receptor Cascade | MyD88 deficiency (TLR2/4) | R-HSA-5602498 | 2.40E-08 |
| Toll-like Receptor Cascade | IRAK4 deficiency (TLR2/4) | R-HSA-5603041 | 4.04E-08 |
| TNF Signaling | TNFR2 non-canonical NF-kB pathway | R-HSA-5668541 | 1.17E-07 |
| T-cell Signaling | TCR signaling | R-HSA-202403 | 1.47E-07 |
| T-cell Signaling | Phosphorylation of CD3 and TCR zeta chains | R-HSA-202427 | 2.40E-07 |
| T-cell Signaling | Downstream TCR signaling | R-HSA-202424 | 4.99E-07 |
| Homeostasis | Integrin cell surface interactions | R-HSA-216083 | 6.17E-07 |
| Apoptosis | Caspase activation via Death Receptors in the presence of ligand | R-HSA-140534 | 6.17E-07 |
| Antigen processing-Cross presentation | Antigen Presentation: Folding | R-HSA-983170 | 8.49E-07 |
| Toll-like Receptor Cascade | MyD88:MAL(TIRAP) cascade initiated on plasma membrane | R-HSA-166058 | 6.17E-07 |
| Toll-like Receptor Cascade | Toll Like Receptor TLR6:TLR2 Cascade | R-HSA-168188 | 6.17E-07 |

**Supplementary Table 4.** Top 30 significant pathways associated with AMR on whole-transcriptome. Enriched pathways are retrieved from Reactome repository, we grouped redundant categories in accordance with physiological function associated. Adjusted -values (referred as q- values) depict significant enrichment from WT gene set.

| **General Class** | **Top 30 network all genes** | **Reactome identifier** | **q-value** |
| --- | --- | --- | --- |
| Innate immune system | Neutrophil degranulation | R-HSA-6798695 | 1.51E-20 |
| Adaptive Immune System | Immunoregulatory interactions between a Lymphoid and a non-Lymphoid cell | R-HSA-198933 | 1.31E-14 |
| Interferon Signaling | Interferon gamma signaling | R-HSA-877300 | 5.38E-14 |
| Interferon Signaling | Interferon Signaling | R-HSA-913531 | 1.93E-09 |
| Interleukin Signaling | Interleukin-10 signaling | R-HSA-6783783 | 1.98E-09 |
| Interleukin Signaling | Signaling by Interleukins | R-HSA-449147 | 3.25E-09 |
| Interferon Signaling | Interferon alpha/beta signaling | R-HSA-909733 | 1.33E-06 |
| Endothelial activation | Cell surface interactions at the vascular wall | R-HSA-202733 | 3.48E-06 |
| Antigen processing-Cross presentation | Antigen processing-Cross presentation | R-HSA-1236975 | 5.94E-06 |
| Apoptosis | Caspase activation via Death Receptors in the presence of ligand | R-HSA-140534 | 6.53E-06 |
| Homeostasis | Platelet activation | R-HSA-76002 | 3.06E-05 |
| Toll-like Receptor Cascade | Toll-like Receptor Cascades | R-HSA-168898 | 3.11E-05 |
| Apoptosis | Caspase activation via extrinsic apoptotic signalling pathway | R-HSA-5357769 | 3.60E-05 |
| Apoptosis | Regulated Necrosis | R-HSA-5218859 | 8.59E-05 |
| Toll-like Receptor Cascade | IRAK4 deficiency (TLR2/4) | R-HSA-5603041 | 1.26E-04 |
| Toll-like Receptor Cascade | Toll Like Receptor 4 (TLR4) Cascade | R-HSA-166016 | 1.27E-04 |
| Apoptosis | Programmed Cell Death | R-HSA-5357801 | 1.50E-04 |
| Signal Transduction | GPVI-mediated activation cascade | R-HSA-114604 | 1.50E-04 |
| Interleukin Signaling | Interleukin-4 and Interleukin-13 signaling | R-HSA-6785807 | 2.26E-04 |
| Antigen processing-Cross presentation | ER-Phagosome pathway | R-HSA-1236974 | 2.26E-04 |
| Signal Transduction | FCERI mediated MAPK activation | R-HSA-2871796 | 2.41E-04 |
| Infection | Parasite infection | R-HSA-9664407 | 4.00E-04 |
| Infection | Leishmania phagocytosis | R-HSA-9664417 | 4.00E-04 |
| Infection | FCGR3A-mediated phagocytosis | R-HSA-9664422 | 4.00E-04 |
| Toll-like Receptor Cascade | MyD88 deficiency (TLR2/4) | R-HSA-5602498 | 4.48E-04 |
| Toll-like Receptor Cascade | MyD88:MAL(TIRAP) cascade initiated on plasma membrane | R-HSA-166058 | 4.48E-04 |
| Toll-like Receptor Cascade | Toll Like Receptor TLR6:TLR2 Cascade | R-HSA-168188 | 4.48E-04 |
| Signal Transduction/Infection | Purinergic signaling in leishmaniasis infection | R-HSA-9660826 | 6.09E-04 |
| Signal Transduction/Infection | Cell recruitment (pro-inflammatory response) | R-HSA-9664424 | 6.09E-04 |
| Toll-like Receptor Cascade | Toll Like Receptor TLR1:TLR2 Cascade | R-HSA-168179 | 6.38E-04 |

**Supplementary Figure 1.** Boxplots displaying the intragraft expression changes of the top 30 ranked significant genes associated to the AMR rejection signature derived from the B-HOT panel. AMR-associated gene expression is correlated with the relative antibody-mediated rejection grade from the diagnostic assessment of endomyocardial biopsies included in the study.


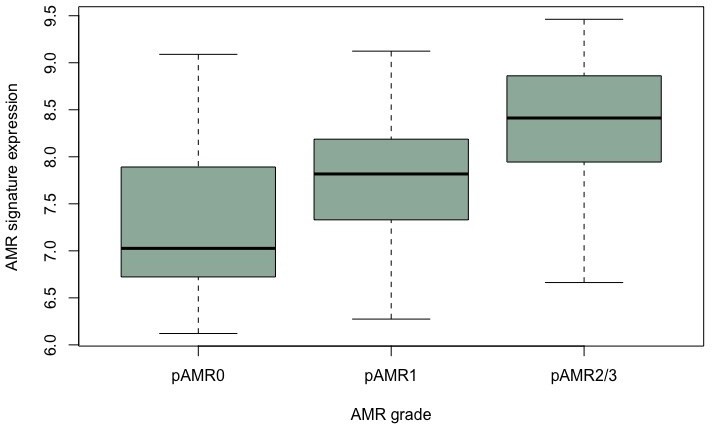


**Supplementary Figure 2.** Hierarchical clustering of enriched term associated with AMR, for B-HOT genes (A) and whole-transcriptome (B). Pathways are gathered according to pairwise similarity of the enriched term calculated. The size of the dots represents the number of genes in the significant differentially expressed gene list associated with the pathway and the color intensity of the dots represents the false discovery rate adjusted p-value.

**A**

**
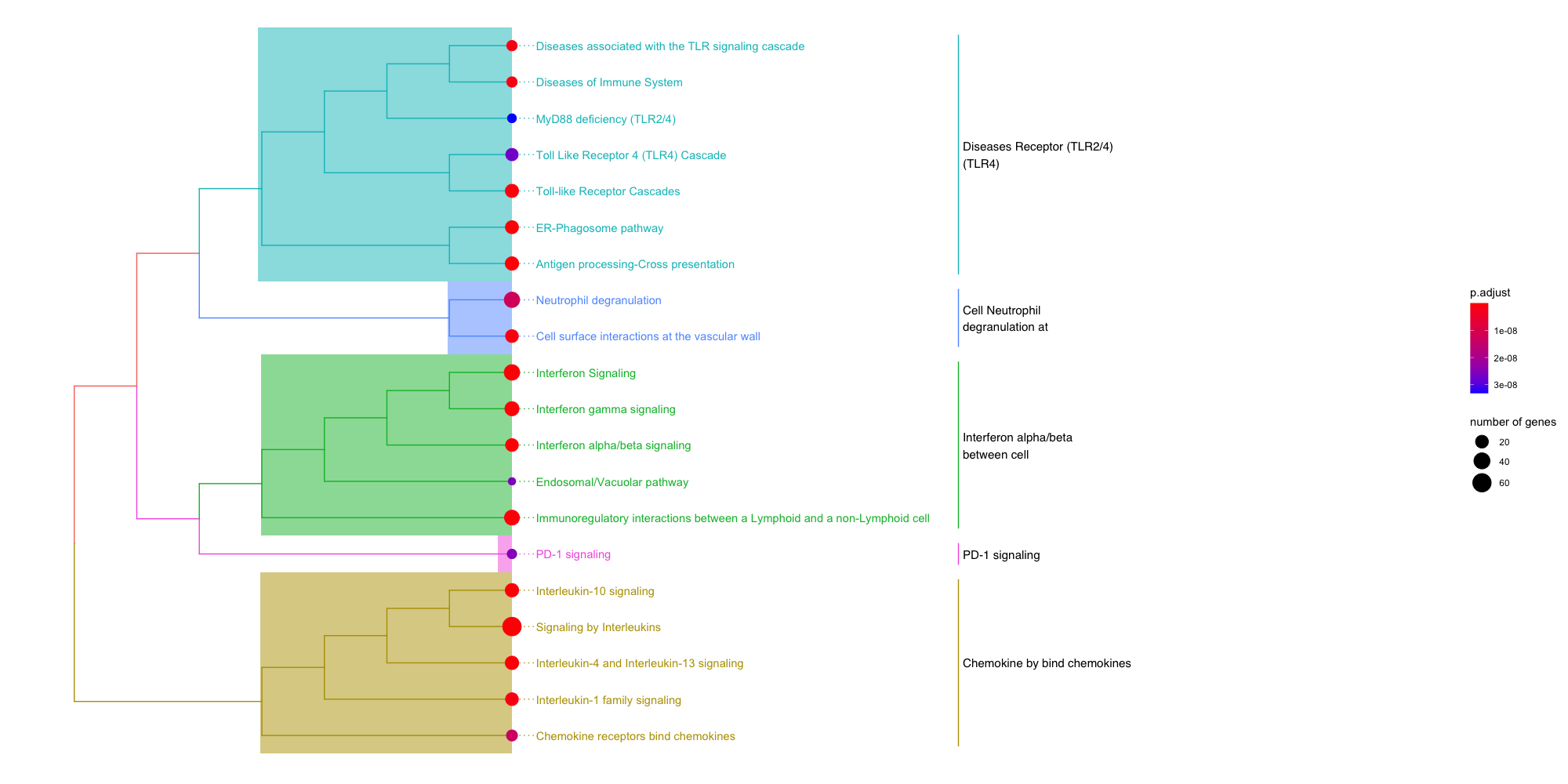
**

**B**

**
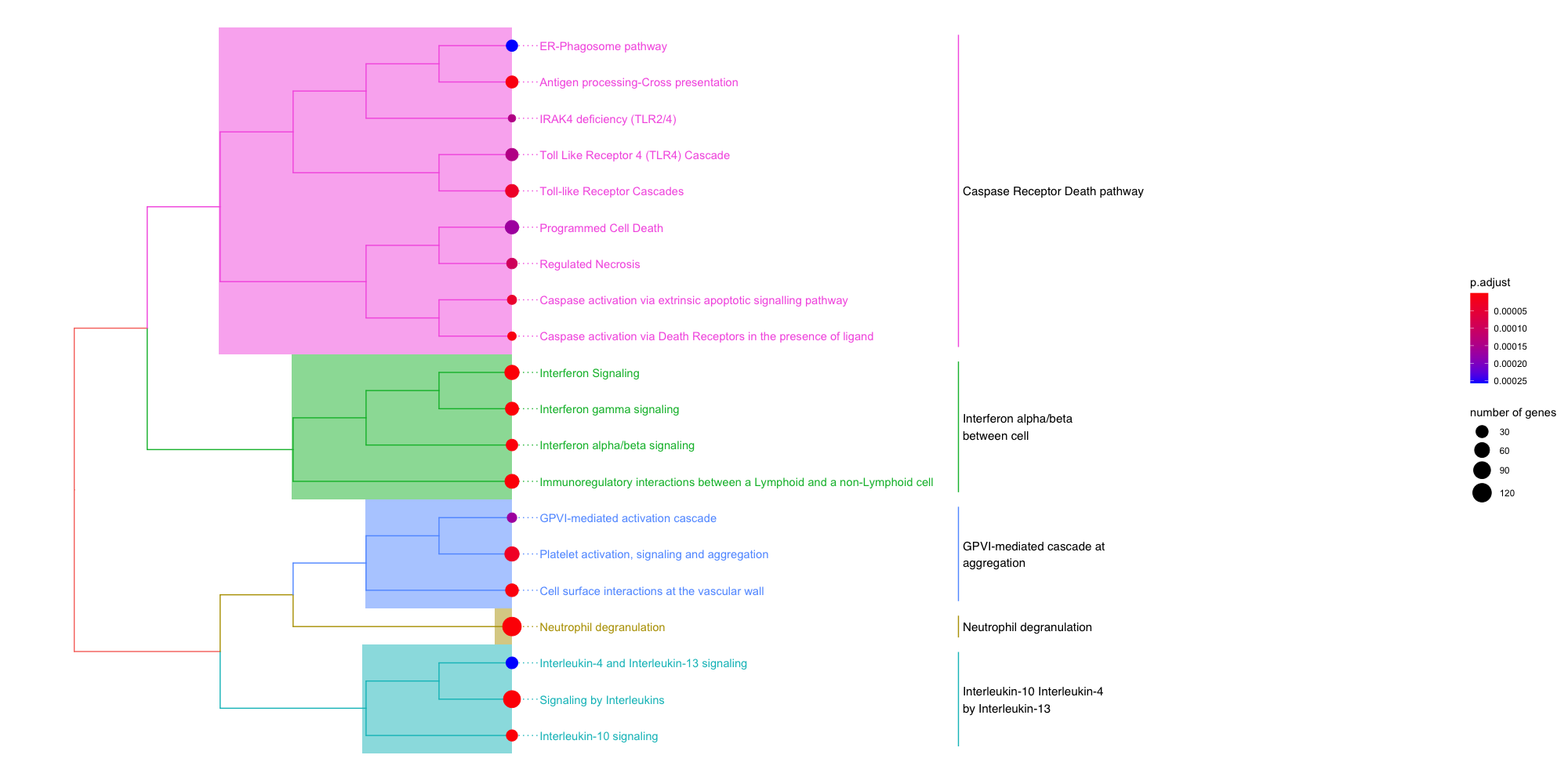
**
